# Kinetics of Chromium(III) Transport Through a Liquid Membrane Containing DNNSA as a Carrier

**DOI:** 10.3390/ijms10030964

**Published:** 2009-03-09

**Authors:** Paweł Religa, Roman Gawroński, Paweł Gierycz

**Affiliations:** 1 Faculty of Material Science, Technology and Design, Technical University of Radom, Chrobrego 27, Radom, Poland; E-Mail: p.religa@pr.radom.pl; 2 Faculty of Chemical and Process Engineering, Warsaw University of Technology, Waryńskiego 1, Warsaw, Poland; E-Mail: gawronski@rekt.pw.edu.pl; 3 Institute of Physical Chemistry, Polish Academy of Sciences, Kasprzaka 44/52, Warsaw, Poland

**Keywords:** Transport kinetics of chromium(III), dinonylnaphthalenesulfonic acid (DNNSA), bulk liquid membrane

## Abstract

Kinetics of Cr(III) ions transport through a bulk liquid membrane containing dinonylnaphthalenesulfonic acid (DNNSA) as a carrier, flowing over aqueous phases, has been examined. Special attention has been paid to the effect of the membrane’s velocity flow on the chromium concentration decrease in a feed phase. For the description of relationships of chromium(III) concentration in particular phases with the time, a model based on the assumption of consecutive first-order reactions was proposed. Satisfactory compatibility of experiments and model results have been obtained both for the membrane flow velocities below 0.0034 m·s^−1^ when the interfaces begin to fluctuate slightly and for low initial Cr(III) concentration in the feed phase.

## 1. Introduction

Liquid membranes (LM) have appeared quite recently as a new prospective separation method. LM soon became very popular in various fields due to their good selectivity and efficiency achieved by the presence of a mobile and selectively acting agent (a carrier). The most popular application of liquid membranes concerns the removal and recovery of metal ions from wastewaters [1–3].

Chromium is one of the most important metals used commonly both in various processes and in production of different products. Because of its high toxicity and limited resources chromium discharges into the environment are strictly regulated.

Simultaneously to the works improving conventional methods of chromium separation intensive research has been conducted on new methods (for example liquid membranes). Most of these studies are focused on chromium(VI) [4–6] because of its hazardous properties. Chromium(III), as a non toxic form, is largely ignored by the most researchers, but a high concentration of chromium(III) not only has a negative influence on living organisms but also oxidizes, in the environment, to chromium(VI) [7,8].

The results of our investigation of the kinetics of Cr(III) ion transport through a bulk liquid membrane containing dinonylnaphthalenesulfonic acid (DNNSA) as a carrier, flowing over aqueous phases, are presented in this paper.

## 2. Experimental Section

**Figure 1.**
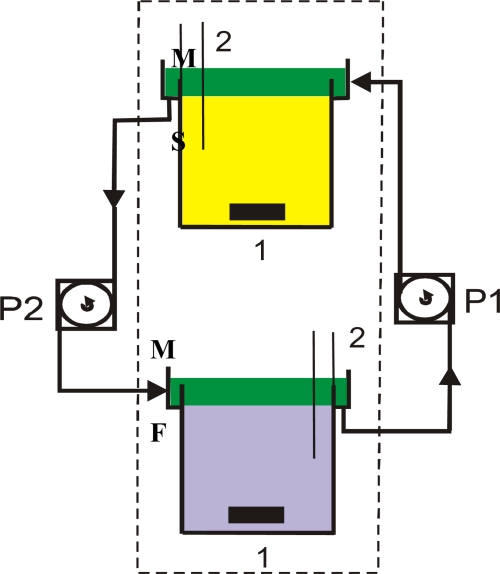
Scheme of the measuring set with flows bulk liquid membrane. 1 – magnetic stirrers, 2 -sampling. M – membrane, F – feed phase, S – stripping phase, P1, P2 –pumps.

All experiments were performed in a system where aqueous phases flow over the membrane. The scheme of the experimental setup is shown in Figure 1.

It consists of two separate Plexiglass vessels containing aqueous phases. The feed phase (F) was a chromium chloride solution (CrCl_3_ analytical grade, POCh) with an initial Cr(III) concentration of C_P_ = 0.019, 0.038 and 0.058 M and pH = 4. The pH was adjusted with 0.1 M NaOH (NaOH analytical grade, Chempur). The stripping phase (S) was an aqueous 4 M H_2_SO_4_ (95% H_2_SO_4_ analytical grade, Chempur) solution. The volumes and the interfaces of both phases were the same and equal: V = 400 cm^3^, F = 56 cm^2^. Aqueous phases were mixed by magnetic stirrers (1) at a rotation rate of 50 rpm.

The liquid membrane (M) was an organic phase containing the dinonylnaphthalenesulfonic acid (DNNSA, Fluka) as a carrier dissolved in a mixture of kerosene and o-xylene. The concentration of DNNSA was C_DNNSA_ = 0.2 mol·dm^−3^. The membrane circulated between the vessels with velocity of flow w = 0.0027, 0.0034 and 0.0049 m·s^−1^. The flow of the liquid membrane was forced by peristaltic pumps (P1, P2) ensuring good mixing of the membrane. For the better flow stability of the membrane the overflow weirs to the vessels were fixed. The physico-chemical parameters of each phase were investigated earlier [9] and in the experiment the optimal ones were used.

The samples of aqueous phases were taken out by sockets (2) and chromium concentration was determined with a SPEKOL 220 spectrophotometer at a wavelength of 595 nm. The corresponding chromium concentration in the membrane was established from the material balance. The measuring setup was placed in thermostat and all experiments were performed at 25 ± 1°C.

## 3. Results and Discussion

### 3.1. Theoretical

The extraction reaction on the feed side (F) interface of a liquid membrane (M) can be written as follows [10]:

(1) $${\text{Cr}}_{\left(\text{F}/\text{M}\right)}^{3+}\;+\;3{\text{RSO}}_{3}{\text{H}}_{\left(\text{M}/\text{F}\right)}\;↔\;{\left({\text{RSO}}_{3}\right)}_{3}{\text{Cr}}_{\left(\text{M}/\text{F}\right)}\;+\;3{\text{H}}_{\left(\text{M}/\text{F}\right)}^{+}$$

and the reaction on the stripping side interface of the liquid membrane in the presence of H_2_SO_4_ assumes the following form:

(2) $${\left({\text{RSO}}_{3}\right)}_{3}{\text{Cr}}_{\left(\text{M}/\text{S}\right)}\;+\;3{\text{H}}_{\left(\text{S}/\text{M}\right)}^{+}\;↔\;{\text{Cr}}_{\left(\text{S}/\text{M}\right)}^{3+}\;+\;3{\text{RSO}}_{3}{\text{H}}_{\left(\text{M}/\text{S}\right)}$$

**Figure 2.**
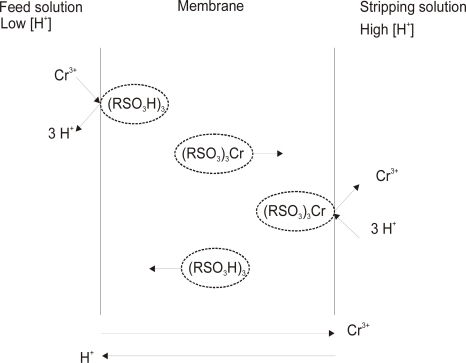
Schematic description of coupled counter transport of Cr(III).

According to these reactions the transport of chromium(III) is coupled by countercurrent transport of H^+^ ions. The mechanism of coupled countercurrent transport of Cr(III) ions through the membrane is schematically presented in Figure 2.

The variation of chromium(III) concentration in individual phases vs. time (as shown in a later Section) suggests that the ion transport obeys the kinetic laws of two consecutive irreversible first-order reactions according to the scheme:

$${\text{Cr(III)}}_{\text{F}}\;\overset{{\text{k}}_{\text{F}}}{\to }{\text{Cr(III)}}_{\text{M}}\;\overset{{\text{k}}_{\text{S}}}{\to }\;{\text{Cr(III)}}_{\text{S}}$$

where: Cr(III)_F_, Cr(III)_M_, Cr(III)_S_ – are the chromium(III) ions in the feed, membrane and stripping phases, respectively, and k_F_, k_S_ - are the pseudo-first-order apparent membrane entrance and exit rate constants, respectively.

It is evident that the presence of the proton gradient drives the whole transport process to completion without any back leakage of chromium(III) ions.

Using the reduced concentrations: ${\text{R}}_{\text{F}}=\frac{{\text{C}}_{\text{F}}}{{\text{C}}_{\text{p}}}$, ${\text{R}}_{\text{M}}=\frac{{\text{C}}_{\text{M}}}{{\text{C}}_{\text{p}}}$, ${\text{R}}_{\text{S}}=\frac{{\text{C}}_{\text{S}}}{{\text{C}}_{\text{p}}}$, where: C_F_, C_M_, C_S_ - chromium concentration appropriate in a feed, membrane and strip-ping phase, C_p_ – initial chromium concentration in a feed phase;

The above kinetic scheme may be described by the following set of equations [11]:

(3) $$\frac{{\text{dR}}_{\text{F}}}{\text{dt}}\;=\;-{\text{k}}_{\text{F}}\;\cdot \;{\text{R}}_{\text{F}}$$

(4) $$\frac{{\text{dR}}_{\text{M}}}{\text{dt}}\;=\;{\text{k}}_{\text{F}}\;\cdot \;{\text{R}}_{\text{F}}\;-\;{\text{k}}_{\text{S}}\;\cdot \;{\text{R}}_{\text{M}}$$

(5) $$\frac{{\text{dR}}_{\text{S}}}{\text{dt}}\;=\;{\text{k}}_{\text{S}}\;\cdot \;{\text{R}}_{\text{M}}$$

Since k_F_ ≠ k_S_, after integration of those equations, we get:

(6) $${\text{R}}_{\text{F}}\;=\;\text{exp}.(-{\text{k}}_{\text{F}}\;\cdot \;\text{t})$$

(7) $${\text{R}}_{\text{M}}\;=\;\frac{{\text{k}}_{\text{F}}}{{\text{k}}_{\text{S}}\;-\;{\text{k}}_{\text{F}}}\cdot [\text{exp}.(-{\text{k}}_{\text{F}}\;\cdot \;\text{t})\;-\;\text{exp}.(-{\text{k}}_{\text{S}}\;\cdot \;\text{t})]$$

(8) $${\text{R}}_{\text{S}}\;=\;1-\;\frac{1}{{\text{k}}_{\text{S}}\;-\;{\text{k}}_{\text{F}}}\cdot [{\text{k}}_{\text{S}}\;\cdot \;\text{exp}.(-{\text{k}}_{\text{F}}\;\cdot \;\text{t})-{\text{k}}_{\text{F}}\;\cdot \;\text{exp}.(-{\text{k}}_{\text{S}}\;\cdot \;\text{t})]$$

R_F_ decreases mono-expotentially with time, whereas the time variation of both R_M_ and R_S_ is bi-exponential. Additionally R_M_ has a maximum (for dR_M_/dt = 0) which allows for calculation of maximum values of time and chromium concentration in the membrane, according to the following equations:

(9) $$({\text{C}}_{\text{M}}{)}_{\text{max}}\;=\;{\text{C}}_{\text{p}}\;\cdot \;{\left(\frac{{\text{k}}_{\text{F}}}{{\text{k}}_{\text{S}}}\right)}^{\frac{{\text{k}}_{\text{S}}}{{\text{k}}_{\text{S}}-{\text{k}}_{\text{F}}}}$$

(10) $${\text{t}}_{\text{max}}\;=\;\frac{\text{ln}\frac{{\text{k}}_{\text{S}}}{{\text{k}}_{\text{F}}}}{{\text{k}}_{\text{S}}\;-\;{\text{k}}_{\text{F}}}$$

Differentiating the eqs. (6–8) the entrance (J_F_) and exit (J_S_) fluxes may be calculated. They are described by the following equations:

(11) $${\text{J}}_{\text{F}}\;=\;\frac{{\text{V}}_{\text{F}}}{{\text{F}}_{\text{F}/\text{M}}}\;\cdot \;{\text{k}}_{\text{F}}\;\cdot \;{\text{C}}_{\text{p}}\;\cdot \;\text{exp}(-{\text{k}}_{\text{F}}\;\cdot \;\text{t})$$

(12) $${\text{J}}_{\text{S}}\;=\;\frac{{\text{V}}_{\text{S}}}{{\text{F}}_{\text{M}/\text{S}}}\cdot \;\frac{{\text{k}}_{\text{F}}}{{\text{k}}_{\text{S}}-{\text{k}}_{\text{F}}}\;\cdot \;{\text{C}}_{\text{M}}\;\cdot \;(\text{exp}(-{\text{k}}_{\text{F}}\;\cdot \;\text{t})-\text{exp}(-{\text{k}}_{\text{S}}\;\cdot \;\text{t}))$$

At t = 0, when the concentration of chromium in the feed phase is equal to the initial concentration (C_F_ = C_P_) the flux J_F_ assumes the following form:

(13) $${\text{J}}_{\text{F}}\;=\;\frac{{\text{V}}_{\text{F}}}{{\text{F}}_{\text{F}/\text{M}}}\;\cdot \;{\text{k}}_{\text{F}}\;\cdot \;{\text{C}}_{\text{P}}$$

The value of the flux J_S_ at t = t_max_, when the concentration of chromium in the membrane reaches its maximum (C_M_ = C_max_), can be calculated from the equation:

(14) $${\text{J}}_{\text{S}}\;=\;\frac{{\text{V}}_{\text{S}}}{{\text{F}}_{\text{M}/\text{S}}}\;\cdot \;{\text{k}}_{\text{S}}\;\cdot \;({\text{C}}_{\text{M}}{)}_{\text{max}}$$

### 3.2. Effect of the velocity of the membrane’s flow

**Figure 3.**
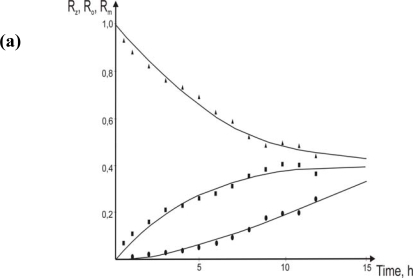

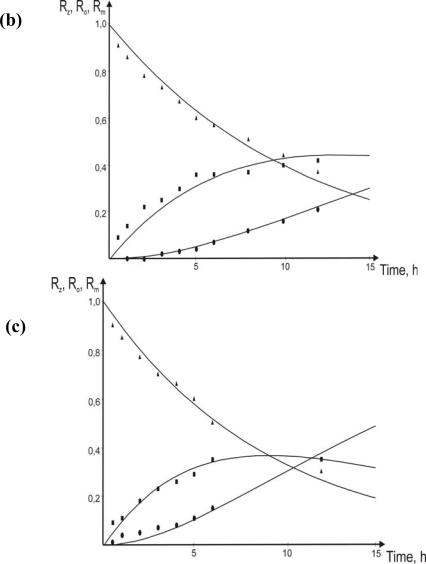
Changes of chromium(III) concentration in particular phases with time for three different flow velocity of the membrane: (a) w = 0.0027 m·s^−1^, (b) w = 0.0034 m·s^−1^, **(c)** w = 0.0049 m·s^−1^and initial chromium concentration *C_p_* = 5.8·10^−5^ mol·m^−3^(▴- R_Z_, ▪ - R_m_, • – R_S_, 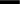 - eqs.6–8).

Changes of chromium(III) concentration in particular phases with time for different flow velocities of membrane and its comparison with theoretical prediction are shown in Figure 3.

The transport of Cr(III) should involve three basic routines: (1) – the mass transport in the “bulk” phase (convection via stirring), (2) – the mass transport near the interface (the diffusion across the diffusion layer) and (3) – the interfacial reaction. The effect of the mass transport in the “bulk” phase can be neglected if the stirrer rotation speed of the aqueous phases is high enough and thus the bulk concentration of Cr(III) is uniform every time. It is also expected that a stronger mixing can decrease the thickness of the diffusion layer and thus increase the kinetics reliance on the interfacial chemical reaction.

**Figure 4.**
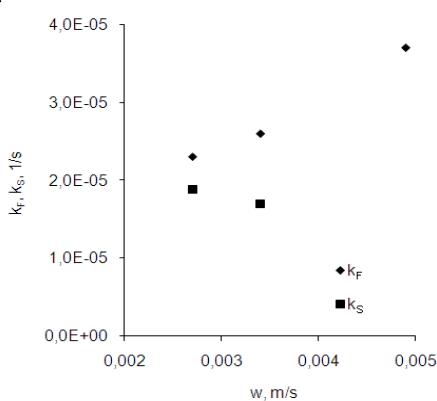
The pseudo-first-order apparent membrane entrance (k_F_) and exit (k_S_) rate constants for different membrane flow velocity (initial chromium concentration *C_p_ =* 5.8·10^−5^ mol·m^−3^).

Figure 4 indicates that the flow velocity of the membrane affects transport rates of the Cr(III) ions through the membrane. The constant k_F_ increases nearly linearly with the increase of the membrane flow velocity for the investigated range of velocity. It suggests that the diffusion is the limiting step in the feed-membrane transport of Cr(III). The constant k_S_ is nearly constant for the investigated range of the velocity. These results show that the chemical reaction is the limiting step in the membrane-stripping transport of chromium(III) ions.

**Figure 5.**
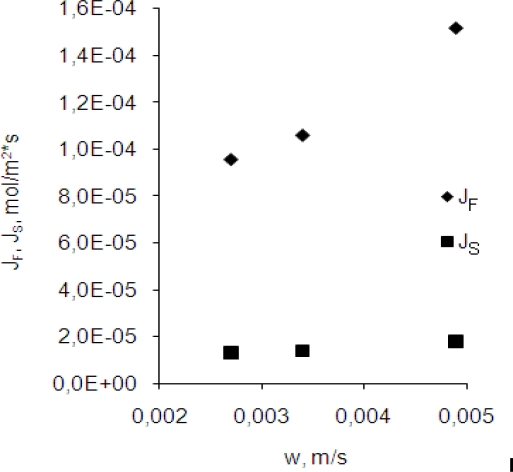
Variation of the entrance (J_F_) and exit (J_S_) fluxes with membrane flow velocity for initial chromium concentration *C_p_* = 5.8·10^−5^ *mol*·*m*^−^*^3^*.

For all cases the stripping of chromium(III) was established as a limiting step of the process (k_S_ < k_F_). With the increase in differences between transport kinetics of chromium(III) on both sides of the membrane an increase in chromium concentration in the membrane was observed. During almost the same time interval the increase in chromium concentration in the membrane was higher for the faster flow over the membrane. Increase of chromium concentration in the membrane caused an increase of maximum entrance (J_F_) flux which is presented in Figure 5. The results presented indicate that a flow of the membrane over the liquid phases with velocity close to 0.0034 m·s^−1^ had favorable influence on efficiency of a transport of chromium(III) in the studied system. Higher flow velocity of membrane caused instabilities of the liquid-liquid interfaces.

### 3.3. Effect of initial chromium(III) concentration in the feed phase

**Figure 6.**
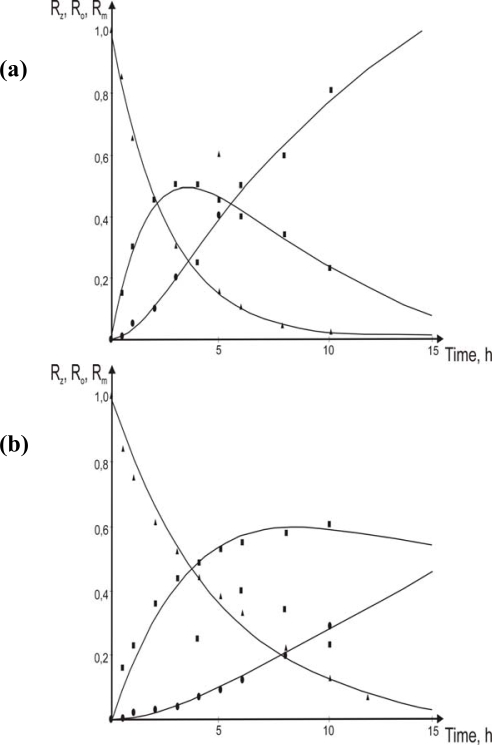

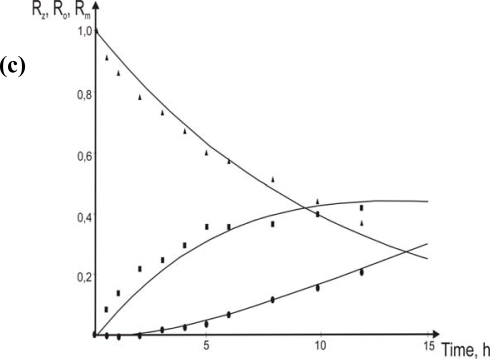
Variation of chromium(III) concentration at individual phases with time for a different initial chromium(III) concentration into the feed phase: (a) 1.9·10^−2^ mol·dm^−3^; (b) 3.8·10^−2^ mol·dm^−3^, (c) 5.8·10^−2^ mol·dm^−3^. Velocity of the membrane’s flow w = 0.0034 m·s^−1^ (▴ - R_Z_, ▪ - R_m_, • – R_S_, 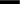 - eqs.6–8).

The results of experimental data demonstrated variation of chromium concentration in the individual phases in the system with the flow of the membrane over aqueous phases vs. time for different initial chromium(III) concentration in the feed phase are in an agreement with a theoretical prediction for the velocity of the membrane equal w = 0.0034 m·s^−1^ (Figure 6).

Decrease in initial chromium concentration in the feed phase causes high increase in extraction efficiency. For initial chromium concentration equal C_P_ = 1.9·10^−5^ *mol*·*m*^−3^ the high degree of chromium extraction (92%) was reached during a short time of ca. 5 hours. Additionally at the lower initial chromium(III) concentration in the feed phase the sharply growth of Cr(III) ions concentration into the stripping phase was observed. Moreover the low chromium concentration and a mixing of the membrane caused by its flow counteracted the accumulating of chromium inside the membrane.

**Figure 7.**
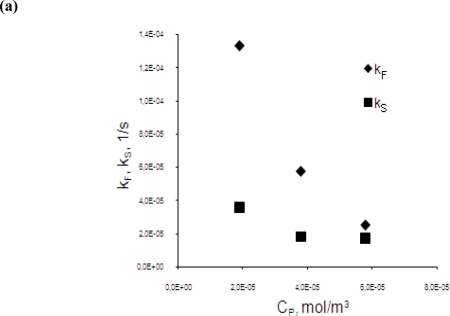

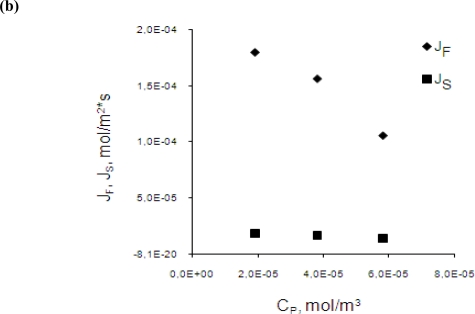
Variation of: (a) the pseudo-first-order apparent membrane entrance (k_F_) and exit (k_S_) rate constants and, (b) the entrance (J_F_) and exit (J_S_) fluxes with initial chromium concentration in the feed phase for membrane flow velocity w = 0.0034 m·s^−1^.

As shown in Figure 7, the increase in initial chromium concentration in the feed phase clearly caused a reduction of the chromium extraction rate from the feed to the membrane phase. The rate of stripping is changed insignificantly. As a result the ratio of extraction and stripping rates decreases. But the stripping is still a limitation step of the process in the investigated range of initial chromium(III) concentrations in the feed phase.

**Figure 8.**
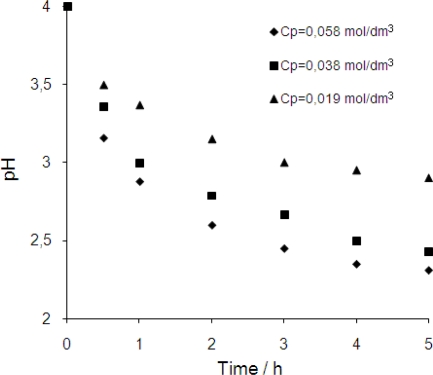
Variation of the feed phase pH with time of experiment.

There are some reasons of that. The hydrolyzed form of chromium(III) is extracted better by complexing agent [9] but the hydrolysis level decreases with an increase in initial chromium(III) concentration in the solution [12]. Additionally, during the first hour of the process, a sharp pH decrease was observed in the feed phase (Figure 8).

This was a consequence of the high proton gradient drive and this is in accordance with equation (1). In the solution with low pH, below 2, chromium(III) exists only in the Cr^3+^ form [12]. Probably this chromium(III) form is much less attractive for the DNNSA-carrier than hydrolyzed forms of chromium(III). The kinetics of process drop as a consequence. Moreover, as suggested by Coelhoso *et al.* [13] because tangential surface tension acts in the system it is possible for water to protrude into the organic phase, facilitating H^+^ ion transport across the membrane. This reduces the driving force and causes a sharp decrease in the process rate, so transport of chromium proceeded in two stages as has been presented earlier [14]. The existence of aqueous protrusions does not mean that the liquid membrane has collapsed. It was not possible because the thickness of the organic phase was equal to ca. 10 mm.

## 4. Conclusions

For the description of dependence of chromium(III) concentration in particular phases vs. time, in the system with the flowing liquid membrane, a model based on the assumption of consecutive first-order reactions was proposed. Satisfactory compatibility of experiments and model results were stated for cases when the velocity of the membrane flow was in the range of 0.0027 – 0.0049 m/s and the initial chromium(III) concentration in the feed phase was below *C_F_* = 3.8·10^−5^ m·s^−1^. Decrease in the velocity of the membrane-flow and increase in the initial chromium(III) concentration in the feed phase caused decrease in the rate and efficiency of the ion transfer. Additionally, the discrepancy between the experimental data and theoretical predictions increased.
